# Late-onset ovarian hyperstimulation syndrome developing during ovarian stimulation in an ectopic pregnancy: a case report

**DOI:** 10.1186/s13256-020-02439-0

**Published:** 2020-07-20

**Authors:** Haipeng Huang, Yasushi Takai, Kouki Samejima, Tatsuya Narita, Shunichiro Ichinose, Yukiko Itaya, Yoshihisa Ono, Shigetaka Matsunaga, Masahiro Saitoh, Kazunori Baba, Naoki Hayashi, Hiroyuki Seki

**Affiliations:** 1 Department of Obstetrics and Gynecology, Saitama Medical Center, Saitama Medical University, 1981 Kamoda, Kawagoe, Saitama, 350-3550 Japan; 2 Women’s Clinic Fujimino, Fujimi, Japan

**Keywords:** OHSS, Laparoscopic surgery, Ectopic pregnancy, P4 levels, Controlled ovarian stimulation (COS)

## Abstract

**Background:**

Ovarian hyperstimulation syndrome is normally induced by ovarian stimulation drugs. Severe cases of ovarian hyperstimulation syndrome involve complications such as renal failure and thrombosis. Evidence has recently been developed for a method to prevent ovarian hyperstimulation syndrome. Most cases of ovarian hyperstimulation syndrome are of an early-onset type, which occurs shortly after injection of human chorionic gonadotropin. However, late-onset ovarian hyperstimulation syndrome, which occurs in a pregnancy cycle, also requires caution. We report our experience in treating a woman who was transported to our hospital with a severe case of ovarian hyperstimulation syndrome occurring during ovarian stimulation and who was determined to have an ectopic pregnancy.

**Case presentation:**

Assisted reproductive technology was planned for a 29-year-old nulligravida Japanese woman diagnosed with bilateral fallopian tube obstruction and right-sided hydrosalpinx. On day 1 of controlled ovarian stimulation, the result of her human chorionic gonadotropin urine test was negative, and her serum levels of luteinizing hormone, estradiol, and progesterone were normal. On day 11 of controlled ovarian stimulation, the levels of estradiol and progesterone had risen to 9679 pg/ml and 16 ng/ml, respectively, prompting suspension of controlled ovarian stimulation. Eleven days after controlled ovarian stimulation was suspended, the patient demonstrated ascites that did not improve despite administration of cabergoline, and she was transported to our hospital 2 days after. Late-onset ovarian hyperstimulation syndrome suggested that she was pregnant, and her serum human chorionic gonadotropin level was 27,778 IU/ml. She underwent laparoscopic bilateral salpingectomy and was diagnosed with right tubal pregnancy.

**Conclusion:**

In an ectopic pregnancy, human chorionic gonadotropin sometimes increases later than in an intrauterine pregnancy. In our patient’s case, endogenous human chorionic gonadotropin following the start of controlled ovarian stimulation may have caused late-onset ovarian hyperstimulation syndrome. The key to early detection of similar cases may be to suspect pregnancy in the event of unexpectedly high progesterone levels during ovarian stimulation.

## Background

Ovarian hyperstimulation syndrome (OHSS) is typically caused by ovarian stimulation drugs [1, 2]. Severe cases of OHSS involve complications such as renal failure and thrombosis. Evidence has recently been established for a method to prevent OHSS [3]. Most cases of OHSS are early-onset OHSS, which occurs shortly after injection of human chorionic gonadotropin (hCG). However, late-onset OHSS, which occurs in a pregnancy cycle, also requires caution.

Late-onset OHSS occurs 12–17 days after injection of hCG and develops in a pregnancy cycle in 96.7% of cases [4]. According to the literature, in multiple pregnancies, hCG levels are high, and OHSS can become severe [4]. Some OHSS cases caused by ectopic pregnancy have also been reported [5].

We present a case of a patient with an ectopic pregnancy in the context of late-onset OHSS. In an ectopic pregnancy, the level of hCG production is lower than in a normal pregnancy at the same gestational age, so early diagnosis by an hCG urine test was impossible at the start of ovarian stimulation in our patient. We also suggest that the assessment of elevated progesterone (P4) may have been necessary to suspect pregnancy in our patient’s case.

## Case presentation

Our patient was a 29-year-old, 161-cm-tall Japanese woman weighing 62 kg, with a body mass index of 23.9 kg/m^2^. She had previously undergone an induced abortion at age 20 years and had received conservative therapy with methotrexate for left tubal pregnancy at age 27 years. She had been a housewife since she had married at age 25 years, had no other medical history, and had taken no medications. She did not like smoking or alcohol.

Infertility-related testing at her previous infertility clinic revealed that her antimüllerian hormone level was 2.65 ng/ml, and her basal levels of estradiol (E2), luteinizing hormone (LH), follicle-stimulating hormone (FSH), and prolactin were 16.2 pg/ml, 2.1 mU/ml, 5.1 mU/ml, and 11.7 ng/ml, respectively. Her thyroid-stimulating hormone (TSH) level was 0.86 μIU/ml, and her menstrual cycle was 28 days. She did not demonstrate any ultrasonographic findings characteristic of polycystic ovary syndrome. Hysterosalpingography revealed bilateral tubal obstruction with right-sided hydrosalpinx. The patient was scheduled to undergo assisted reproduction for 1.5 years of secondary infertility. Salpingectomy was discussed and planned in case of repeated implantation failure.

In blood taken on day 1 of the patient’s last menstrual cycle, her levels of E2, LH, and P4 were 26 pg/ml, 4.4 mIU/ml, and 0.23 ng/ml, respectively. After a negative result was confirmed in an hCG urine test, she was started on oral dydrogesterone 20 mg/day and began daily self-injection of urinary FSH 300 IU on the same day. In blood taken on day 9 of ovarian stimulation, her levels of E2, LH, and P4 were 4569 pg/ml, 1.35 mIU/ml, and 3.5 ng/ml, respectively. Therefore, the urinary FSH was changed to human menopausal gonadotropin 300 IU, which contains high levels of LH. On day 11 of ovarian stimulation, her levels of E2, LH, and P4 were 8679 pg/ml, 0.1 mIU/m, and 16.3 ng/ml, respectively, prompting suspension of ovarian stimulation. The patient had no symptoms during the controlled ovarian stimulation (COS), and no abnormal ultrasound finding was detected during COS.

Eleven days after ovarian stimulation was suspended, the patient had abdominal distension and demonstrated ascites extending to the upper abdomen under ultrasonography. She was diagnosed with OHSS and started on cabergoline 0.5 mg/day and aspirin 100 mg/day, but these failed to improve her condition. Therefore, 13 days after ovarian stimulation was suspended, she was transported to our hospital for intensive care for severe OHSS.

Upon arrival, the patient perceived abdominal distension as well as a soft abdomen, lower abdominal pain, and low back pain during palpation. Her blood pressure, heart rate, body temperature, and oxygen saturation were 113/88 mmHg, 100 beats/minute, 37.0 °C, and 99%, respectively. Her blood test results were as follows: white blood cell count 19,800/μl, hemoglobin 14.2 g/dl, hematocrit 41.3%, C-reactive protein 1.2 mg/dl, total protein 5.1 g/dl, albumin 2.7 g/dl, aspartate transaminase 31 U/L, alanine transaminase 23 U/L, lactate dehydrogenase 210 U/L, sodium 130 mEq/L, potassium 4.4 mEq/L, creatinine 0.48 mg/dl, and uric acid (UA) 5.8 mg/dl. The results of serologic testing for hepatitis B, hepatitis C, and syphilis were negative. Ultrasonography revealed bilateral ovarian enlargement (right ovary length, 8.6 cm; left ovary length, 5.5 cm) as well as ascites extending to the upper abdomen. A chest x-ray showed that both costophrenic angles were sharp, and no pleural effusion was observed. On the basis of these findings, the patient was diagnosed with severe OHSS and hospitalized for further care. Suspicion of late-onset OHSS in a pregnancy cycle prompted measurement of her serum hCG level, which was 27,778 mIU/ml.

When the patient was asked again about her medical history, she stated that her menstrual cycle occurred 28 days prior to the beginning of ovarian stimulation and that she had last had sexual intercourse 16 days prior to the beginning of ovarian stimulation. Thus, at the start of ovarian stimulation, at 4 weeks, 2 days (after her true last menstrual period), bleeding that was originally assumed to be menstruation was deemed to be abnormal uterine bleeding in early pregnancy. When she was transported to our hospital, she was 7 weeks, 3 days pregnant.

**Fig. 1:**
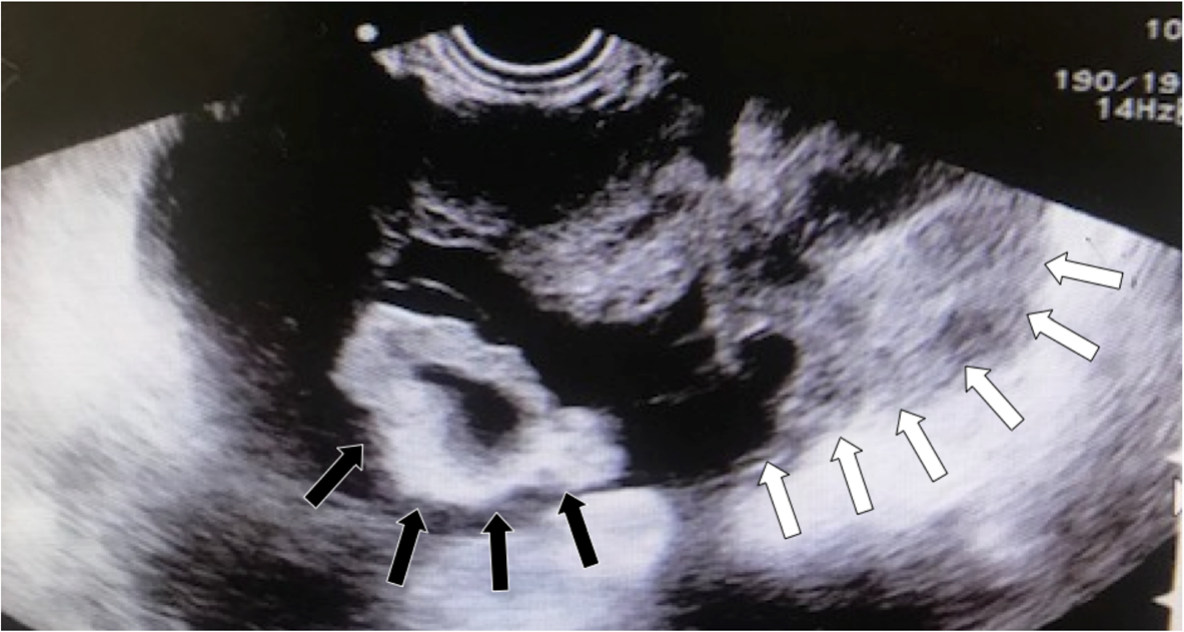
Findings by transvaginal ultrasonography. The *white arrows* indicate the enlarged right ovary; the *black arrows* indicate the fallopian tube, which was believed to have enlarged due to the adjacent hollow structure

A second ultrasound revealed a hollow structure on the lateral aspect of the ovary (Fig. 1) but did not show an embryo inside the ovary. In addition, the corpus luteum was indistinct due to enlargement of the ovary. On the basis of these findings, emergency laparoscopic surgery was performed for suspected right tubal pregnancy.

We performed bilateral laparoscopic salpingectomy, and chorionic villi were macroscopically observed in the right fallopian tube. We also observed approximately 5000 ml of light-yellow ascites. We prevented postoperative thrombus with oral administration of aspirin 100 mg/day and intermittent pneumatic leg compression. We also administered additional oral administration of cabergoline 0.5 mg/day until OHSS improved. On day 4 postoperation, the patient was discharged after demonstrating improvement of ascites, improvement of hemoconcentration, and a favorable reduction in serum hCG (751 mIU/ml). Oral administration of aspirin and cabergoline and aspirin was continued until day 11 postoperation. Her serum hCG level returned to negative on day 24 postoperation, and she resumed her infertility treatment at her previous infertility clinic 3 months after the surgery.

**Fig. 2:**
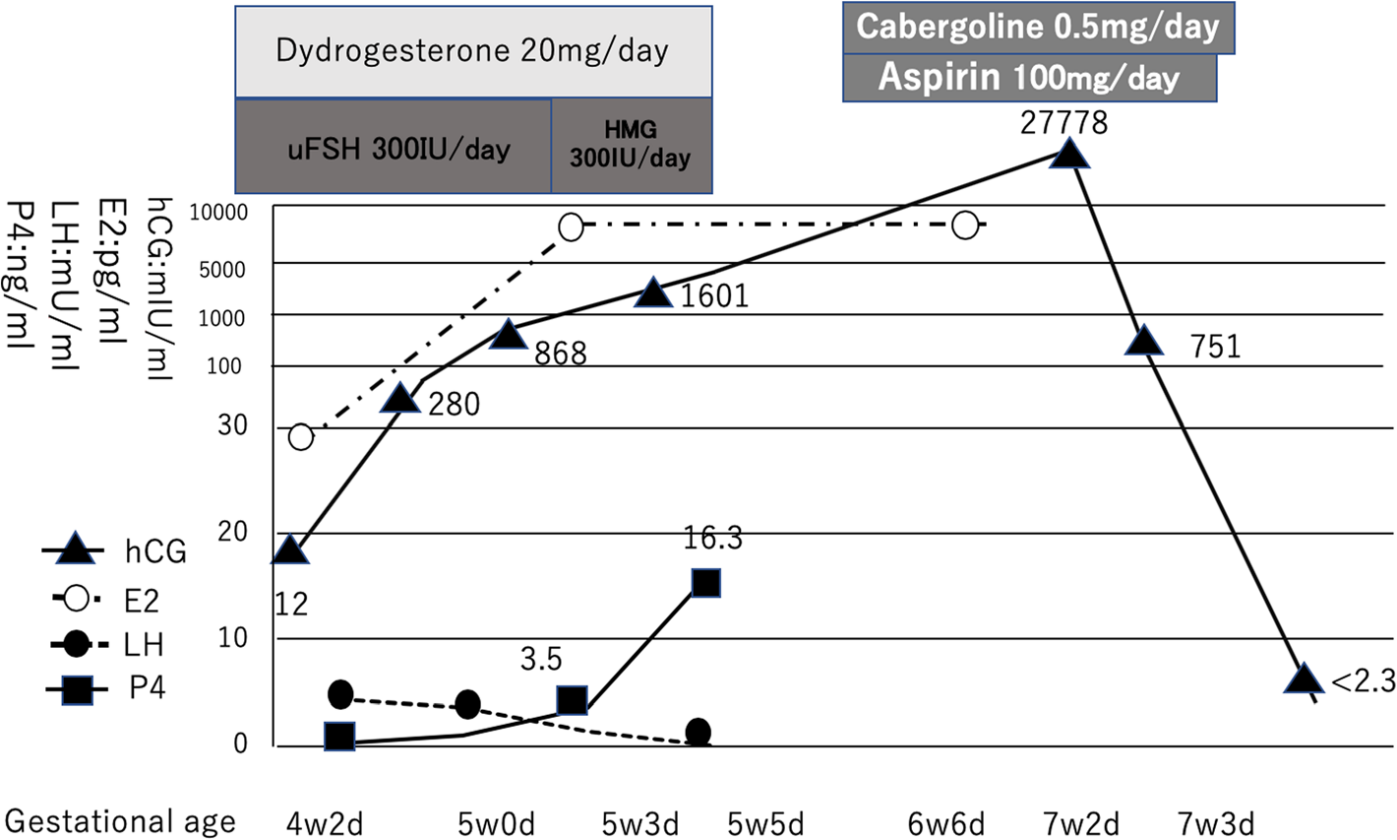
Changes in serum levels of luteinizing hormone, estradiol, progesterone, and human chorionic gonadotropin (hCG). The serum hCG level was tested also in a residual serum sample taken at the referring hospital. Ovarian stimulation had been started by the patient’s previous physician on day 1 of uterine bleeding (after a negative result was confirmed in an hCG urine test). However, the patient was later considered to have been 4 weeks, 2 days pregnant at that point

Her pathology result was determined to be right ampullary tubal pregnancy. We performed a repeated serum hCG test on a specimen of blood preserved by the patient’s previous physician. According to this test, the patient’s serum hCG level at the start of ovarian stimulation was 12 mIU/ml. The patient’s clinical course is shown in Fig. 2.

## Discussion

We treated a rare case in which a patient scheduled to undergo assisted reproduction for tubal factor infertility became pregnant naturally immediately prior to ovarian stimulation and developed late-onset OHSS during ovarian stimulation. We believe that the present case is instructive for physicians conducting infertility treatment. In our patient’s case, we did not observe any evident risk factors for OHSS other than peak E2 level > 3500 pg/ml [6].

The main point in the present case appears to be that the pregnancy was noticed following the start of ovarian stimulation. In a retrospective examination of the case, the following points stood out:

1. At the start of ovarian stimulation (gestational age 4 weeks, 2 days), E2 was 26 pg/ml, P4 was 0.2 ng/ml, and hCG (as measured in a residual serum sample) was 12.0 mIU/ml. Thus, P4 was not elevated, and basal body temperature was believed to be in the low temperature phase. In an ectopic pregnancy, the level of hCG production is lower than in a normal pregnancy at the same gestational age [7], whereas the cutoff value in an hCG urine test is 25 mIU/ml. Therefore, we may not have noticed the pregnancy without serum hCG.
2. Elevated P4 (5 weeks, 3 days): At day 9 of ovarian stimulation, P4 had increased to 3.5 ng/ml and subsequently increased to 16.3 ng/ml at day 11, which should have been interpreted as a sign of pregnancy. But at the previous infertility clinic, the high P4 level was only considered as an irregular process like premature luteinization because of bilateral tubal obstruction with right-sided hydrosalpinx and a negative urine hCG test result at the beginning of COS.
3. Exacerbation of late-onset OHSS (6 weeks, 6 days): Despite ovarian stimulation having been suspended 10 days prior, the patient demonstrated elevated levels of E2 and P4. It may have been necessary to suspect pregnancy in late-onset OHSS.

It may be suggested that an ectopic pregnancy should be suspected when an unexpected P4 rise is observed with no LH elevation during ovarian stimulation.

## Conclusion

In an ectopic pregnancy, hCG sometimes increases later than in an intrauterine pregnancy, so early diagnosis for ectopic pregnancy by an hCG urine test may be impossible at the start of ovarian stimulation. In our patient’s case, endogenous hCG following the start of COS may have caused late-onset OHSS. The key to early detection of similar cases may be to suspect pregnancy in the event of unexpectedly high P4 levels during ovarian stimulation.

### Limitations

The serum hCG values used in this case presentation were measured in a residual serum sample taken at the referring hospital after the diagnosis of ectopic pregnancy. Therefore, these values may be considered reference values.

## Data Availability

Not applicable.

## Abbreviations

COS: Controlled ovarian stimulation
E2: Estradiol
FSH: Follicle-stimulating hormone
hCG: Human chorionic gonadotropin
LH: Luteinizing hormone
OHSS: Ovarian hyperstimulation syndrome
P4: Progesterone
TSH: Thyroid-stimulating hormone
